# MicroRNAs in Cerebrospinal Fluid as Potential Biomarkers for Parkinson’s Disease and Multiple System Atrophy

**DOI:** 10.1007/s12035-016-0253-0

**Published:** 2016-11-14

**Authors:** Tainá M. Marques, H. Bea Kuiperij, Ilona B. Bruinsma, Anouke van Rumund, Marjolein B. Aerts, Rianne A. J. Esselink, Bas R. Bloem, Marcel M. Verbeek

**Affiliations:** 1 Department of Neurology, Radboud University Medical Center and Donders Institute for Brain, Cognition and Behaviour, Nijmegen, The Netherlands; 2 Department of Laboratory Medicine, Radboud University Medical Center, P.O. Box 9101, Nijmegen, 6500 HB The Netherlands; 3 Parkinson Center Nijmegen, Nijmegen, The Netherlands

**Keywords:** Parkinson’s disease, Multiple system atrophy, Biomarkers, microRNA, Cerebrospinal fluid

## Abstract

Parkinson’s disease (PD) and multiple system atrophy (MSA) are both part of the spectrum of neurodegenerative movement disorders and α-synucleinopathies with overlap of symptoms especially at early stages of the disease but with distinct disease progression and responses to dopaminergic treatment. Therefore, having biomarkers that specifically classify patients, which could discriminate PD from MSA, would be very useful. MicroRNAs (miRNAs) regulate protein translation and are observed in biological fluids, including cerebrospinal fluid (CSF), and may therefore have potential as biomarkers of disease. The aim of our study was to determine if miRNAs in CSF could be used as biomarkers for either PD or MSA. Using quantitative PCR (qPCR), we evaluated expression levels of 10 miRNAs in CSF patient samples from PD (*n* = 28), MSA (*n* = 17), and non-neurological controls (*n* = 28). We identified two miRNAs (miR-24 and miR-205) that distinguished PD from controls and four miRNAs that differentiated MSA from controls (miR-19a, miR-19b, miR-24, and miR-34c). Combinations of miRNAs accurately discriminated either PD (area under the curve (AUC) = 0.96) or MSA (AUC = 0.86) from controls. In MSA, we also observed that miR-24 and miR-148b correlated with cerebellar ataxia symptoms, suggesting that these miRNAs are involved in cerebellar degeneration in MSA. Our findings support the potential of miRNA panels as biomarkers for movement disorders and may provide more insights into the pathological mechanisms related to these disorders.

## Introduction

Parkinson’s disease (PD) is the second most common neurodegenerative disorder in elderly people, characterized by the loss of dopaminergic neurons, resulting in a movement disorder with symptoms such as postural instability, bradykinesia, rest tremor, and rigidity [1]. Non-motor symptoms are also reported, such as neuropsychiatric dysfunction, cognitive impairment, sleep disorders, olfactory dysfunction, sensory symptoms, and pain [2]. Multiple system atrophy (MSA), a rare, rapidly progressive, and very debilitating disease, is characterized by parkinsonism or cerebellar ataxia, in combination with autonomic dysfunction. Furthermore, both diseases are part of the spectrum of α-synucleinopathies, characterized by accumulation of the protein α-synuclein in Lewy bodies as the major neuropathological hallmark. Due to the overlap of symptoms with PD at early stages, MSA may be misdiagnosed as PD. At later stages, MSA has, however, a distinct disease progression and usually poor response to anti-Parkinson treatment [3].

The diagnoses of PD or MSA are based on extensive clinical and neurological evaluations, cerebral MRI, and response to anti-Parkinson treatment [4, 5]. Biomarkers that specifically classify patients with PD and discriminate them from other atypical parkinsonism, such as MSA, are not yet available. A reliable biomarker to complement clinical diagnoses would be very useful preferably at early stages of diseases to avoid misdiagnoses, provide adequate disease management and patient counseling, and for research purposes.

Cerebrospinal fluid (CSF) is the body fluid which is closest to the brain, and therefore, it is the most promising body fluid for identification of biomarkers for neurodegenerative disorders. Several proteins have been investigated in CSF as biomarkers for PD and atypical parkinsonism disorders, such as neurofilament light chain (NFL), tau, α-synuclein, and Aβ42 [6]. NFL and total tau protein levels discriminate MSA from PD with reasonable accuracy [7–9], whereas CSF α-synuclein is reduced in all α-synucleinopathies and Aβ42 was investigated as an indicator of cognitive decline (reviewed in [10]).

MicroRNAs (miRNA or miR) have been investigated and suggested as biomarkers in a large variety of diseases, including neurodegenerative disorders [11]. They are small non-coding RNAs, of approximately 20 nucleotides, that act in post-transcriptional regulation of messenger RNA (mRNA), blocking the translation into proteins by binding to the 3′ untranslated region (3′UTR). A single miRNA can downregulate the expression of hundreds of genes, and in turn, each mRNA could be controlled by numerous miRNAs [12].

In the past years, the discovery of circulating miRNAs in body fluids caught the attention of researchers due to the opportunity to use them as biomarkers of disease [13]. Since then, in the field of neurodegeneration, they have been studied in brain tissue, serum, plasma, blood, and CSF [14].

Several miRNAs have been identified at abnormal levels in PD and MSA and suggested as potential biomarkers. For example, miR-34b/c [15], miR-133b [16], and miR-205 [17] were found at lower levels in brain tissue from PD patients compared to controls. miR-19a/b [18–20] and miR-30c [18, 21] showed lower expression levels either in PD or MSA in CSF, serum, and peripheral blood mononuclear cells (PBMC), while concentrations of miR-132 [20] were higher in CSF of PD compared to controls. In addition, miR-24 and miR-148b were found at altered levels in serum of PD and MSA patients [21].

The aim of our study was to determine if these miRNAs could be used as disease-specific biomarkers for either PD or MSA when quantified in CSF.

## Methods

### Cerebrospinal Fluid Samples from Patients and Controls

Our patients were selected from a previous longitudinal study at the Radboud University Medical Center (Nijmegen, the Netherlands), which included patients referred to our tertiary movement disorder center between January 2003 to December 2006 [22]. These patients were followed for 3 years, and the final diagnosis for each patient was established by two neurologists who were experts in movement disorders based on the current criteria for PD [5] and MSA [4]. The disease severity was evaluated using the Hoehn and Yahr (H&Y) scores [23], the Unified Parkinson’s Disease Rating Scale (UPDRS) [24], the International Cooperative Ataxia Rating Scale (ICARS) [25], and the Mini-Mental State Examination (MMSE) [26]. An overview of the patients’ details is given in Table 1.

**Table 1:** Patient group characteristics

	Control	PD	MSA	*p* value^a^
Number	28	28	17	
Gender (men/women)	15/13	21/7	13/4	*p* = 0.15
Age at inclusion (years)	62.9 ± 8	54.5 ± 10.4	62.5 ± 9.7	*p* = 0.002
Disease duration (months)	NA	38.9 ± 40.2	25.7 ± 14.5	*p* = 0.66
Follow-up (years)	NA	*n* = 24	*n* = 11	NA
		4.8 ± 2.1	3.1 ± 1.2	
Disease severity				*p* value^b^
H&Y score	NA	*n* = 27	*n* = 17	*p* = 0.05
		1.8 ± 0.6	2.1 ± 0.8	
UPDRS score	NA	*n* = 26	*n* = 17	*p* = 0.17
		25.1 ± 14.3	29.2 ± 11.7	
ICARS score	NA	*n* = 26	*n* = 14	*p* < 0.0001
		1.9 ± 3.2	9.9 ± 7.5	
MMSE score	NA	*n* = 27	*n* = 16	*p* = 0.79
		28.1 ± 1.8	27.6 ± 3.2	

Values are expressed as mean ± standard deviation

*n* number of samples, *PD* Parkinson’s disease, *MSA* multiple system atrophy, *NA* not applicable, *H&Y* Hoehn and Yahr score, *UPDRS* Unified Parkinson’s Disease Rating Scale, *ICARS* International Cooperative Ataxia Rating Scale, *MMSE* Mini-Mental State Examination

^a^Parameters were analyzed with ANOVA using Bonferroni’s post hoc test, except for gender, which was analyzed using chi-squared test

^b^Comparison between PD and MSA was performed using Student’s *t* test or Mann-Whitney *U* test

The control group consisted of patients aged above 50, which resembles the age when symptoms of PD or MSA are usually observed. These patients had been evaluated by the Neurology Department for suspicion of neurological disorders but turned out not to have a neurological disease after extensive investigation.

For selection of CSF samples, we adhered to the following criteria: leukocyte number count fewer than 5 cells/μl and erythrocyte number fewer than 200 cells/μl to avoid blood contamination of CSF, since we previously observed that the presence of blood cells in CSF affects miRNA levels [27–29]. CSF samples from PD, MSA, and non-neurological controls were collected in polypropylene tubes, centrifuged, aliquoted, and stored at −80 °C until further analysis. All participants provided written informed consent.

### RNA Isolation, Reverse Transcription, and Quantitative PCR

CSF samples from PD (*n* = 28), MSA (*n* = 17), and non-neurological controls (*n* = 30) were randomly distributed in three groups for the procedures of RNA isolation, reverse transcription into cDNA, pre-amplification, and quantitative PCR (qPCR) as previously described by our group [27, 28]. In addition, three samples were used as an inter-plate control for qPCR reactions to determine inter-plate variation in miRNA quantification.

Our selection of miRNAs was based on previous publications on these miRNAs, in which they were proposed as potential biomarkers for PD or MSA, either in body fluids or in brain tissue. We also took into consideration that the predicted targets of these selected miRNAs should include genes that had been previously linked to PD or MSA. For this, we submitted our miRNA selection to the target prediction program TargetScan version 7 [30], including results of all conserved and poorly conserved sites, and for a second confirmation of target prediction, we submitted the selection to the microT-CDS software from the DIANA online platform [31, 32], with a settled threshold of 0.7 (suggested by the software for optimal accuracy on target prediction). An overview of the predicted targets is listed in Table 2.

**Table 2:** Number of predicted targets and specification of targets linked to PD/MSA for each miRNA

MicroRNA	Prediction software			
	TargetScan		DIANA	
	Number of predicted targets	Targets already linked to PD or MSA	Number of predicted targets	Targets already linked to PD or MSA
miR-19a-3p	3968	PARK2, LRRK2, VPS35	1261	PARK2
miR-19b-3p	3968	PARK2, LRRK2, VPS35	1262	PARK2
miR-24-3p	6215	ATP13A2, VPS35	978	ATP13A2, EIF4G1
miR-30c-5p	4304	LRRK2, DNAJC13	1670	LRRK2, DNAJC13
miR-34b-3p	4165	SNCA, PARK2, VPS35	928	SNCA
miR-34c-5p	4374	SNCA, PLA2G6, SLC1A4	894	–
miR-132-5p	1230	–	54	–
miR-133b	2976	SNCA	1050	SNCA, DNAJC13
miR-148b-3p	4011	SNCA, PARK2, PARK7, VPS35, HTRA2, SLC1A4	903	SNCA, PARK7
miR-205-5p	4413	LRRK2, HTRA2, SQSTM1	1371	LRRK2

*SNCA* synuclein alpha, *ATP13A2* ATPase 13A2, *VPS35* retromer complex component, *SQSTM1* sequestosome 1, *SLC1A4* solute carrier family 1 member 4, *PLA2G6* phospholipase A2 group VI, *PARK7* parkinsonism-associated deglycase, *PARK2* parkin RBR E3 ubiquitin protein ligase, *LRRK2* leucine-rich repeat kinase 2, *HTRA2* HtrA serine peptidase 2, *EIF4G1* eukaryotic translation initiation factor 4 gamma 1, *DNAJC13* DnaJ heat shock protein family (Hsp40) member C13

The selected miRNAs for this study were as follows: hsa-miR-19a-3p, hsa-miR-19b-3p, hsa-miR-24-3p, hsa-miR-30c-5p, hsa-miR-34b-3p, hsa-miR-34c-5p, hsa-miR-132-5p, hsa-miR-133b, hsa-miR-148b-3p, and hsa-miR-205-5p. In addition, has-miR-16-5p and U6 small nuclear RNA (snRNA) were used as reference RNAs. The primer sequences from Applied Biosystems (Foster City, CA, USA) can be found at http://www.appliedbiosystems.com.

### Data Analysis

The miRNA expression levels were normalized to the geometric mean (GM) [27] of the cycle threshold (Ct) values of two small reference RNAs in each sample, i.e., miRNA-16 and U6. The Ct values of these two small reference RNAs were similar in the three groups. To calculate the relative expression levels (RELs), we first calculated the GM by the formula$GM=\sqrt{Ct\left[miR16\right]\times Ct\left[\mathrm{U}6\right]}$ and then calculated the difference between the Ct values of the miRNA target and the GM (∆Ct = CtmiRNA − GM). Finally, we calculated the relative expression by REL = 2^−ΔCt^.

Data were analyzed using GraphPad Prism, version 5 (La Jolla, CA, USA), and IBM SPSS Statistics 22 (Armonk, NY, USA). D’Agostino and Pearson omnibus normality test was used to check data distribution. For comparison among the three groups, in the case of parametric data, one-way analysis of variance (ANOVA) was used with Bonferroni’s post hoc test, and for non-parametric data, Kruskal-Wallis test with Dunn’s post hoc test for multiple comparisons. For comparison between PD and MSA, Student’s *t* test or Mann-Whitney *U* test was selected for parametric and non-parametric distributed data, respectively. Analyses of covariance (ANCOVA) were performed to control for possible confounding factors that could influence miRNA expression level, such as age.

Analysis of receiver operator characteristic (ROC) was performed to assess the diagnostic accuracy of parameters. The Youden index was used to determine the optimal cutoff values. To test if a combination of miRNAs could improve differentiation between groups, binary logistic regression analysis was applied including all miRNAs and a model was created for each pair of comparison, which was subjected to ROC curve analysis for test of accuracy.

We also performed a correlation analysis of the miRNAs in the three groups and also between miRNA expression levels in PD or MSA and clinical parameters (H&Y, UPDRS, ICARS, MMSE); for all analyses, we used Spearman’s rho test.

## Results

### MiRNA Expression in CSF

We evaluated the expression levels of ten miRNAs (miR-19a, miR-19b, miR-24, miR-30c, miR-34b, miR-34c, miR-132, miR-133b, miR-148b, and miR-205) in CSF of patients diagnosed with PD or MSA and non-neurological controls. In accordance to our selection criteria, CSF leukocyte and erythrocyte number did not differ between groups. Gender distribution was equal in the three groups, but age was significantly different among groups, due to the inclusion of relatively young patients in the PD group. The parameters used for evaluation of disease severity, H&Y, UPDRS, and MMSE scores were similar between PD and MSA, except for the ICARS score, which is expected since cerebellar ataxia is prominently observed in MSA and not in PD. A summary of these parameter details is shown in Table 1.

The selected reference RNAs could be detected in all samples with the exception of two samples (both from the control group). Failure to quantify these small RNAs is probably due to improper sample processing, despite careful execution of our protocols, and therefore, we excluded these samples from further analysis. The mean Ct values of miRNA-16 (control = 23.6, PD = 22.1, MSA = 22.1) and U6 (control = 26.9, PD = 26.2, MSA = 26.2) were similar in the three groups (ANOVA *p* = 0.90, *p* = 0.06, respectively; data not shown).

**Fig. 1:**
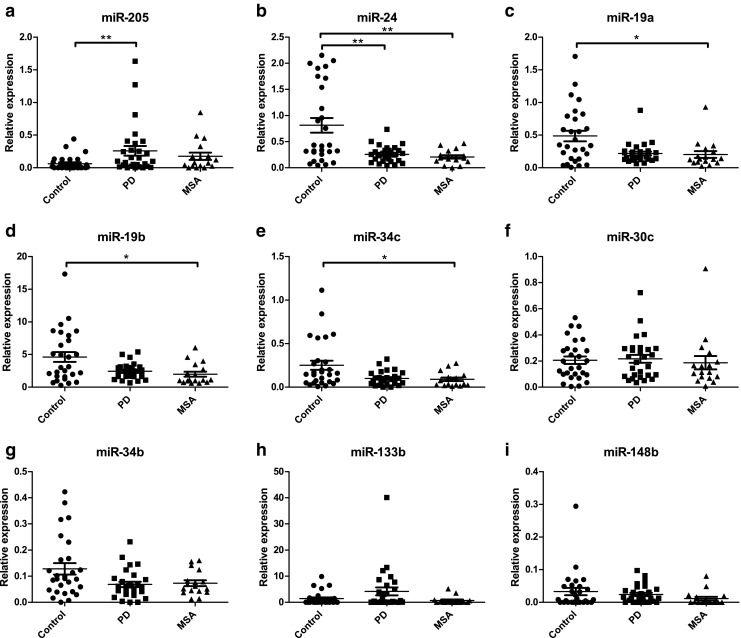
Relative expression values of miRNAs in CSF from controls, PD, and MSA patients. MiR-205 (**a**) and miR-24 (**b**) were able to discriminate PD from non-neurological controls. Lower levels of miR-24 (**b**), miR-19a (**c**), miR-19b (**d**), and miR-34c (**e**) compared to controls allowed the discrimination of MSA from control patients. Data were analyzed using ANOVA. *p < 0.05; **p < 0.001

MiR-132 could not be detected in any sample and therefore had to be excluded from further analysis. An overview of the results for the nine remaining miRNA targets is shown in Fig. 1. Our findings indicated that the mean miR-205 levels were upregulated in PD compared to the control group (*p* = 0.0061; Fig. 1a) by a factor of 4.1. In contrast, miR-24 was downregulated in PD by a factor of 3.1 (*p* = 0.0024; Fig. 1b). Four miRNAs showed lower levels in MSA compared to controls, miR-19a (*p* = 0.0216, factor = 2.4), miR-19b (*p* = 0.0261, factor = 2.3), miR-24 (*p* = 0.0024, factor = 3.9), and miR-34c (*p* = 0.0259, factor = 2.8) (Fig. 1b–e). None of the miRNAs was individually able to discriminate PD and MSA. Because of the age difference between the groups, age was included as a possible confounding factor using ANCOVA. This resulted in the loss of significance of the difference for miR-205 (PD versus controls), but the differences for the other targets were retained.

### Diagnostic Value and Panels

**Fig. 2:**
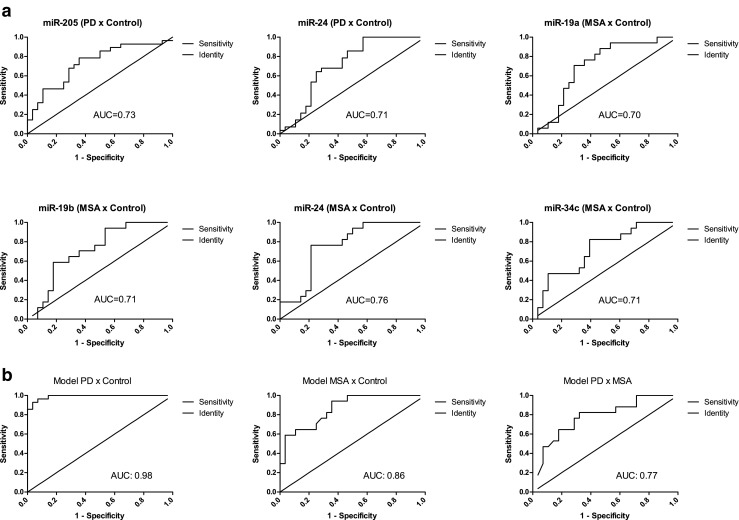
**a** ROC curves of miRNAs with mean levels that were statistically different between patient groups. The compared patient groups are indicated between *brackets*. Areas under the curve (AUC) were 0.70 to 0.76, as indicated. **b** ROC curves of models created from binary logistic regression to improve discrimination between groups. The model created to differentiate PD from controls included miR-19a, miR-19b, miR-24, miR-30c, miR-34b, miR-133b, and miR-205 and resulted in an AUC of 0.98. The model generated for comparison of MSA versus control included miR-24 and miR-205 with an AUC of 0.86. For the model of PD versus MSA, miR-133b and miR-148b were included and showed a moderate value for accuracy with an AUC of 0.77

ROC analysis was performed to determine the diagnostic accuracy of targets that statistically differed between either PD or MSA and controls. The area under the curve (AUC), which indicates the accuracy, was moderate for all targets for discrimination of PD or MSA from controls, with an average AUC of 0.72 (±0.02) (Fig. 2a).

Binary logistic regression analysis was used to investigate if combinations of miRNAs could improve their use as biomarkers. A combination of miRNAs resulted in an improved discrimination of PD from the control group (Fig. 2b) compared to single miRNAs. The created model included miR-19a, miR-19b, miR-24, miR-30c, miR-34b, miR-133b, and miR-205. The AUC from the ROC analysis increased to 0.96 (*p* value <0.0001, sensitivity of 96% and specificity of 92%, cutoff >−0.44, positive likelihood ratio = 13.5), suggesting that the combination of these miRNAs could improve diagnostic accuracy. A similar analysis was performed to generate a model for distinction of MSA from control (Fig. 2b), which included miR-24 and miR-205. With this combination, the diagnostic accuracy increased to an AUC of 0.86 (*p* value <0.0001, sensitivity of 94% and specificity of 64%, cutoff >−1.06, positive likelihood ratio = 2.64). Finally, by using the combination of miR-133b and miR-148b, PD and MSA could be discriminated (Fig. 2b); the ROC analysis showed a moderate value with an AUC of 0.77 (*p* value = 0.001, sensitivity of 82% and specificity of 67%, cutoff >−0.35, positive likelihood ratio = 2.56).

### Correlation

**Fig. 3:**
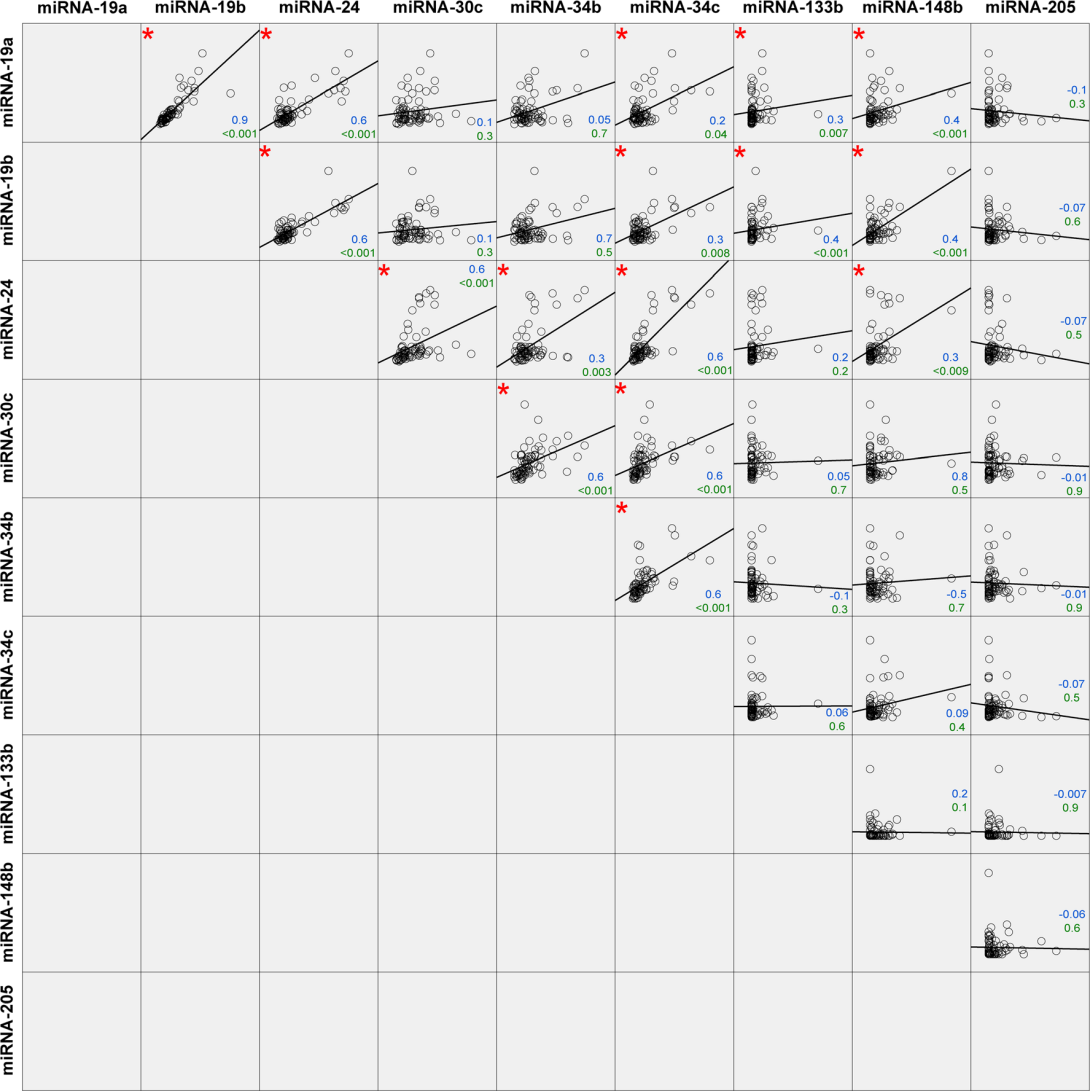
Correlation analysis of all miRNAs among the three disease groups. In total, 16 statistically significant correlations (*p* value below 0.05) were found, indicated with a *red asterisk*. Spearman’s rho coefficient value (upper value (in *blue*)) and *p* value (lower value (in *green*)) are indicated in the graphs

In order to evaluate if there was a correlation between the expression levels of the various miRNAs, we performed a correlation analysis with Spearman’s rho test. We found 16 significant correlations between the miRNAs, and an overview of our findings is shown in Fig. 3. The most prominent correlations were observed between miR-19a, miR-19b, miR-30c, miR-34b, and miR-34c. We also studied correlations between miRNA levels and clinical parameters that were used for evaluation of disease severity, such as the H&Y, UPDRS, ICARS, and MMSE scores. We did not find any correlations in the PD group between the miRNAs and the clinical parameters. However, in the MSA group, we observed the following two negative correlations: miR-24 (*r* = −0.5, *p* = 0.045) and miR-148b (*r* = −0.7, *p* = 0.012), both correlated to ICARS.

## Discussion

The aim of our study was to identify miRNAs that could serve as biomarkers of disease in CSF for either PD or MSA and, ideally, that could discriminate between both diseases. The selection of miRNAs was based on previous publications, in which these miRNAs were identified as potential biomarkers in blood, serum, brain tissue, or CSF. We identified two potential biomarkers for PD (miR-24 and miR-205) and four miRNAs that could be biomarkers for MSA (miR-19a, miR-19b, miR-24, and miR-34c).

### MiRNA Biomarkers for PD

Our results showed an increase of miR-205 in CSF from PD patients when compared to controls, which is in contrast with previous findings in brain tissue of sporadic PD patients [17]. They observed a lower expression of miR-205 in brain regions of 15 patients diagnosed with PD and increased levels of leucine-rich repeat kinase 2 (LRRK2) protein, and this correlation was also confirmed by functional studies with modulation of this miRNA in cell lines and primary neurons in culture. These different observations remain yet unexplained.

We found lower levels of miR-24 in the CSF from both PD and MSA patients as compared to control but no difference between PD and MSA. This is also in contrast to results previously obtained, where miR-24 was found at higher concentrations in PD and MSA serum and increased in MSA in comparison to PD [21]. This discrepancy could be due to the differences in body fluids used (serum versus CSF), where serum levels could represent systemic changes, and CSF is more closely related to neurodegeneration only. The ability of miR-24 to discriminate PD or MSA from healthy controls suggests that it may have potential as a biomarker for α-synucleinopathies. Functional studies would be important to understand the role of miR-24 in the pathology of α-synucleinopathies.

### MiRNA Biomarkers for MSA

In our study, miR-19a was identified at lower levels in MSA compared to control, but no difference was observed between PD and controls. This is not in line with previous findings of decreased levels of miR-19a in serum from PD patients with mutations in the LRRK2 gene compared to healthy controls in a qPCR experiment [19]. This difference could be explained by the absence of PD patients carrying LRRK2 mutation in our patient selection and the difference in body fluids (serum versus CSF). Lower levels of this miRNA were also found in CSF samples of PD in a small RNA sequencing experiment [20]. Unfortunately, neither information about the selection of patients was available in this study nor if the number of erythrocytes or leukocytes in CSF was controlled for, which may interfere with miRNA quantification in CSF [27, 28]. To our knowledge, this is the first time that miR-19a was linked to MSA. Among the predicted targets for miR-19a are the following three important genes already related to PD: PARK2 (parkin), LRRK2, and VPS35 (Table 2), but none of these has previously been linked to MSA, which could be expected due to the little evidence concerning a genetic cause of this disease. Our findings suggest that miR-19a could be a potential biomarker to differentiate MSA from controls, but functional studies for target confirmation and validation in larger cohorts remain necessary.

Similarly to miR-19a, CSF miR-19b levels were lower in MSA compared to controls, which has not previously been reported, although lower levels have also been found in serum of idiopathic PD patients [19]. Lower expression levels in serum were also found in patients with idiopathic rapid eye movement sleep behavior disorder [33], which is often associated to PD, MSA, or Lewy body dementia [34]. Downregulation of this miRNA was also observed in CSF samples and in exosomes isolated from CSF from PD patients compared to controls as observed in a small RNA sequencing experiment [20, 35]. Our study did not reveal a reduction in the concentration of this miRNA in CSF of PD patients, which could be caused by differences in patient selection or differences in the way CSF had been processed or the number of blood cells in the CSF that were included in the previous studies. The similarities in predicted targets of miR-19a/b (see Table 2), the previously published results and our results of lower levels of both miRNAs in MSA (Fig. 1), and the strong correlation between these miRNAs (Fig. 3) all suggest that this miRNA family plays an important role in PD and MSA pathophysiology, but validation in larger cohorts remains necessary to obtain a better understanding of the role of this miRNA in MSA or PD.

In our data, we observed lower levels of miR-34c in CSF from MSA patients. Interestingly, it has been described before that miR-34b and miR-34c concentrations were lower in various brain regions from PD patients [15]. The same group also found decreased brain levels of DJ-1 and parkin proteins, which are both tightly linked to PD. A previous study identified that the p53 protein may activate the miR-34 family (reviewed in [36]), and in addition, it has been suggested that the α-synuclein, DJ-1, and parkin proteins may inhibit p53 activity [37–39]. Functional studies in human dopaminergic cells confirmed that mRNA of α-synuclein is a target of action of miR-34b/c and that its inhibition leads to α-synuclein aggregation [40]. The involvement of miR-34b/c in PD and MSA is not completely understood but it is a potential therapeutic target, and their use as a biomarker requests a confirmation in larger cohorts.

### Detection of Other miRNAs in CSF

We found similar levels of miR-30c, miR-133b, and miR-148b in CSF samples of PD, MSA, and control. Our findings disagree with previous descriptions of reduced levels of miR-30c in PD serum [21] and peripheral blood mononuclear cell [18], decreased levels of miR-133b in PD brain tissue [16], and lower levels of miR-148 in PD serum and increased levels in MSA serum [21]. At the moment, the only explanation for this discrepancy is the difference in sample types.

In general, we were not able to retrieve information on the number of erythrocytes and leukocytes in the CSF samples studied in any of the previous publications, in which specific miRNAs were suggested as potential biomarkers. We would like to stress the importance to exclude CSF samples contaminated by blood since this may affect CSF miRNA levels when future studies are undertaken to evaluate the potential of miRNAs to serve as a biomarker of diseases [27, 28]. We also recommend attention to storage time and centrifugation of samples and use of geometric means for normalization of the data to avoid possible bias [29].

### MiRNA Panels

Since each individual miRNA had limited value to serve as a biomarker for either PD or MSA, given the relatively low AUC, we analyzed if combinations of miRNAs could increase the diagnostic accuracy. By applying binary logistic regression analysis including all miRNAs, we created models for each pair of comparison. This resulted in the definition of combinations of miRNAs that differentiated either PD (AUC = 0.96) or MSA (AUC = 0.86) from controls at high accuracy level and PD from MSA at moderate AUC (0.77). Hence, our findings allow us to suggest three different panels of miRNAs to be used as biomarkers for the distinction of PD, MSA, and controls. However, confirmation in independent cohorts will be necessary for final establishment of their diagnostic power.

### Correlations of miRNAs with Other Parameters

Interestingly, we noted similarities in the predicted targets (Table 2) of the various miRNAs that ended up in the regression models. The genes PARK2, LRRK2, and VPS35 appeared as possible targets in almost all the miRNAs that were enrolled in the model for differentiation of PD from controls. No gene overlap was found in the predicted targets in the model for differentiation of MSA compared to control, but in the model that compared both diseases, α-synuclein was shown to be a target for action of the two miRNAs.

We observed a strong correlation between miR-19a and miR-19b, which was not surprising since they both belong to the same family. Similarly, a strong correlation was observed for miR-34b and miR-34c, which also belong to the same family.

We also studied correlations between miRNAs and clinical parameters. Intriguingly, we found a negative correlation of miR-24 and 148b with the ICARS score in MSA, but not in PD or controls. This score is clinically used for evaluation of cerebellar ataxia, which is a symptom that is predominantly observed in MSA. Therefore, it is possible that genes regulated by miR-24 and 148b may control cerebellar functions and that deregulation of these miRNA levels may contribute to MSA. Further studies will have to confirm this association, however.

### Study Limitations

A few limitations apply to our study. First, unlike in mRNA expression studies, there are no universally accepted reference miRNAs to which target miRNAs can be normalized, since miRNAs are tissue and disease specific. Therefore, we chose to use the geometrical mean of two small RNAs to normalize the data, but other methods and other reference miRNAs have been used as well in other studies.

Second, differences between studies in sample processing may affect the results of miRNA quantification, as (micro)RNA levels are very low in CSF. Other techniques, such as miRNA arrays or small RNA sequencing, offer great possibilities to identify new miRNAs, but are not sensitive enough to detect all miRNAs, whereas qPCR is very sensitive but may yield results with high variability in different studies.

Third, the CSF samples we used were only collected for research purposes creating a selection bias. Therefore, our findings should be validated in larger cohorts and by other centers to confirm the biomarker potential of the miRNAs. Inclusion of other parkinsonian disorders would also yield more detailed insight into the association of specific miRNAs with neurodegenerative movement disorders.

## Conclusion

MiRNAs play an important role in control of gene expression, and their stability in body fluids offers a great opportunity to use them as biomarkers. We identified two miRNAs that were successful in distinguishing PD from controls and four miRNAs that were able to differentiate MSA from controls. Moreover, we also created three panels consisting of a combination of CSF miRNAs that were able to discriminate either PD or MSA from controls and also between both diseases with high to medium accuracy levels. Therefore, these panels of miRNAs may be used as biomarkers of disease.

Furthermore, in the MSA group, we observed a correlation of two microRNAs with the ICARS score, a clinical parameter used for quantification of cerebellar ataxia, a combination of symptoms exclusive to MSA patients. Therefore, we suggest further studies to investigate the role of these miRNAs in control of cerebellar gene expression.

## Abbreviations

AUC: Area under the curve
CSF: Cerebrospinal fluid
Ct: Cycle threshold
GM: Geometric mean
miRNA: microRNA
MSA: Multiple system atrophy
PD: Parkinson’s disease
qPCR: Quantitative PCR
REL: Relative expression
ROC: Receiver operator characteristics
